# Burden of Coronary Artery Disease and Peripheral Artery Disease: A Literature Review

**DOI:** 10.1155/2019/8295054

**Published:** 2019-11-26

**Authors:** Rupert Bauersachs, Uwe Zeymer, Jean-Baptiste Brière, Caroline Marre, Kevin Bowrin, Maria Huelsebeck

**Affiliations:** ^1^Department of Vascular Medicine, Klinikum Darmstadt GmbH, Darmstadt, Germany; ^2^Center of Thrombosis and Haemostasis, University of Mainz, Mainz, Germany; ^3^Klinikum der Stadt Ludwigshafen, Medizinische Klinik B, Ludwigshafen am Rhein, Germany; ^4^Bayer AG, Berlin, Germany; ^5^Creativ-Ceutical, Paris, France; ^6^Bayer PLC, Reading, UK

## Abstract

**Background:**

Atherothrombotic disease, including coronary artery disease (CAD) and peripheral artery disease (PAD), can lead to cardiovascular (CV) events, such as myocardial infarction, stroke, limb ischemia, heart failure, and CV death.

**Aim:**

Evaluate the humanistic and economic burden of CAD and PAD and identify unmet needs through a comprehensive literature review.

**Methods:**

Relevant search terms were applied across online publication databases. Studies published between January 2010 and August 2017 meeting the inclusion/exclusion criteria were selected; guidelines were also included. Two rounds of screening were applied to select studies of relevance.

**Results:**

Worldwide data showed approximately 5–8% prevalence of CAD and 10–20% prevalence of PAD, dependent on the study design, average age, gender, and geographical location. Data from the REACH registry indicated that 18–35% of patients with CAD and 46–68% of patients with PAD had disease in one or more vascular beds. Use of medication to control modifiable CV risk factors was variable by country (lower in France than in Canada); statins and aspirin were the most widely used therapies in patients with chronic disease. Survival rates have improved with medical advancements, but there is an additional need to improve the humanistic burden of disease (i.e., associated disability and quality of life). The economic burden of atherothrombotic disease is high and expected to increase with increased survival and the aging population.

**Conclusion:**

CAD and PAD represent a substantial humanistic and economic burden worldwide, highlighting a need for new interventions to reduce the incidence of atherothrombotic disease.

## 1. Introduction

Atherosclerosis is highly prevalent and forms the underlying pathophysiology for coronary artery disease (CAD), peripheral artery disease (PAD), and cerebrovascular (CvD)/carotid artery disease [5, 6]. This progressive condition is characterized by a diseased endothelium, low-grade inflammation, lipid accumulation, and plaque formation within the intima of the vessel wall [7]. Plaque rupture or erosion can provoke superimposed atherothrombosis and subsequent vessel occlusion, leading to cardiovascular (CV) events, including myocardial infarction (MI), stroke, limb ischemia, and CV death [8]. CAD and PAD share a common pathogenesis and risk factors for development (e.g., smoking, dyslipidemia, hypertension, and diabetes mellitus) [9]. Disease in more than one arterial bed is a marker of advanced diffuse atherosclerosis and is associated with a worse prognosis than single-vessel disease [10]. Prognosis can be improved through secondary prevention measures, with lifestyle changes, medicinal control of modifiable CV risk factors, and the prevention of blood clot formation with antithrombotic therapies [11]. Being the leading cause of mortality and morbidity worldwide, atherothrombotic disease places a substantial medical and economic burden on society [7, 12]. Although the majority of evidence on the burden of atherosclerotic disease comes from high-income countries, it is important to note the increasing burden of atherosclerotic CV disease in low- and middle-income countries, a topic reviewed in more detail by Fowkes et al. [13].

This structured literature review aimed to provide a comprehensive overview of the burden of, and unmet need in, patients with CAD and PAD based on relevant real-world studies and reviews. The geographic scope of the review included Canada, France, Germany, Sweden, the UK, and the USA.

## 2. Methods

The search was implemented in the MEDLINE, MEDLINE In-Process, and Embase databases via the OVID interface and in relevant national and international guidelines portals and healthcare associations/organizations, with defined search terms and inclusion/exclusion criteria applied, to identify studies on CAD and PAD relevant to research on the epidemiology, treatment patterns, risk factors, and humanistic and economic burdens of disease. No language restriction was applied. The search time limit was from January 2010 to August 2017. Of note, for completeness we have included a publication from the Global Burden of Diseases (GBD) study published just after the August 2017 cut-off date [14]. Inclusion/exclusion criteria were structured as per the PICOS criteria (Supplementary [Supplementary-material supplementary-material-1]) and applied first to titles and abstracts then to the full-text article; at each step, an additional reviewer carried out a random check of the selected publications. The selection process can be illustrated using a PRISMA chart.

## 3. Results

Publications selection is detailed in Supplementary [Supplementary-material supplementary-material-1].

### 3.1. Epidemiology

In interpreting epidemiological data, it is important to consider that definitions of disease, patient selection, and study design may have impacted on results. This includes whether estimates focused on diagnosed (usually symptomatic) versus overall (including asymptomatic) prevalence of CAD and PAD.

#### 3.1.1. Coronary Artery Disease

Based on GBD study estimates, the global prevalence of CAD was 154 million in 2016, representing 32.7% of the global burden of CV disease and 2.2% of the overall global burden of disease [14]. Based on data from a national health survey collected from 2009 to 2012, the American Heart Association (AHA) estimated a prevalence of CAD of 15.5 million; therefore, 7.6% of men and 5.0% of women in the USA were living with CAD in this time period [15]. In the ONACI registry in France, the incidence of CAD ranged from ~1% per year among men aged 45–65 years old (slightly higher among women of the same age) to ~4% in patients aged 75–84 years old regardless of sex [16].

#### 3.1.2. Peripheral Artery Disease

Based on 2016 GBD study estimates, the global prevalence of PAD was 120 million, representing 25.6% of the global burden of CV disease and 1.7% of the overall global burden of disease [14]. The 1999–2000 US National Health and Nutrition Examination Survey found that 4.3% of individuals aged ≥40 years old had been diagnosed with PAD based on having an ankle-brachial index (ABI) <0.90. In patients aged ≥70 years old, the prevalence was 14.5% [17]. In a retrospective analysis of US healthcare insurer databases (2003–2008), the mean annual prevalence of PAD was 10.7% and the mean annual incidence 2.4% [18]. A study in Canada confirmed PAD in 8.1% of patients in two prospective study cohorts (*N *= 1459; average age 63.5 years), based on an ABI ≤0.9 [19]. In the 2004 prospective German Epidemiological Trial on Ankle Brachial Index study, 6880 unselected patients (mean age 72.5 years) from primary care were screened for PAD (according to an ABI <0.90), which was detected in 19.8% of male and 16.8% of female patients. PAD patients were characterized by high comorbidity (including diabetes, hypertension, and lipid disorders) including other manifestations of atherothrombotic disease [20]. A recent review of epidemiological data on PAD in Europe highlighted evidence suggesting that ABI correlates with marital and socioeconomic factors, especially in men, with separation/divorce, unemployment, and lower educational achievement associated with a lower ABI [21].

#### 3.1.3. Disease in More Than One Vascular Bed

**Figure 1:**
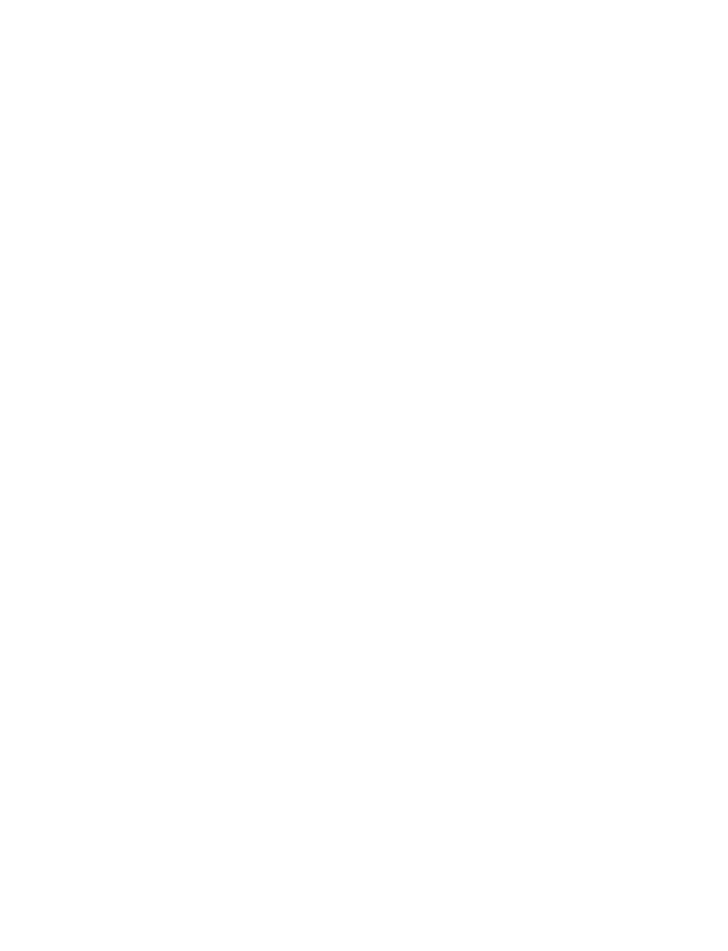
Baseline characteristics and medication use and clinical outcomes in patients with CAD and PAD from the REACH Registries of (a) Canada,^a^ (b) France,^b^ and (c) Germany^c^ [1, 2]. ^a^Patients enrolled from January to October 2004; mean age 68.4 ± 9.9. ^b^Percentage of patients experiencing at least one CV event (Figure (a): CAD* n* = 1356, PAD* n* = 146; Figure (b): CAD *n* = 2397, PAD *n* = 882; Figure (c): CAD *n* = 3328, PAD *n* = 1303). ^c^Patients enrolled from December 2003 to June 2014; mean age 69.1–71.7 across risk groups. ^d^Patients enrolled from December 2003 to June 2014; mean age 68.0–69.4 across risk groups. ACE, angiotensin-converting enzyme; ASA, acetylsalicylic acid; CAD, coronary artery disease; CV, cardiovascular; PAD, peripheral artery disease.

In the REACH datasets, 15.6% of patients enrolled in Canada, 18.2% in France and 27.4% in Germany had established atherothrombotic disease in more than one vascular bed (~18–35% of patients with CAD also had PAD and/or CvD and ~46–68% of patients with PAD also had CAD and/or CvD; mean age ≥68 years across datasets; Figures 1(a)–1(c)) [1, 2]. In another prospective dataset (the GenePAD study, in which patients ≥40 years old with suspected CAD were screened for PAD; mean age 65.5 years), 23.0% of 1186 patients with confirmed CAD or PAD were diagnosed with concomitant disease. Only 7% of patients diagnosed with PAD during the study had a prior PAD diagnosis before enrollment, supporting the notion that PAD is an underdiagnosed condition [22].

### 3.2. Current Treatment Approaches

Clinical guidelines for the management of CAD and PAD have been developed by several societies and organizations [23–32]. The overarching treatment goals in these guidelines are to provide symptom relief (through medication and revascularization), to salvage limbs in cases of PAD, and to prevent future CV events. Recommendations for the secondary prevention of CV events include the control of CV risk factors (e.g., diabetes mellitus, hypertension, and smoking) through lifestyle modification, patient education, and pharmacologic therapy. Use of antithrombotics is also recommended for most patients (Supplementary Tables [Supplementary-material supplementary-material-1] and [Supplementary-material supplementary-material-1]) [23–32]. Several observational studies identified in this literature search provide an insight into current treatment approaches.

#### 3.2.1. Coronary Artery Disease

In data from the global CLARIFY registry, which enrolled outpatients with chronic CAD, patients aged >75 years old were less often treated with beta blockers, lipid-lowering agents, aspirin, or angiotensin-converting enzyme (ACE) inhibitors than younger patients, but were more often treated with calcium channel blockers, angiotensin receptor blockers (ARBs), nitrates, or diuretics (perhaps reflecting the greater burden of comorbidities in older patients) [33]. Furthermore, women were less likely than men to undergo coronary artery angiography or noninvasive tests for myocardial ischemia, as well as percutaneous coronary intervention (PCI) or coronary artery bypass grafting (CABG), or to receive ACE inhibitors or lipid-lowering drugs. The proportion of aspirin use between men and women of all ages was similar [33]. In the REACH registries in Canada, France, and Germany, the majority of patients with CAD were on a statin at enrollment (85.3%, 77.6%, and 79.1%, respectively); the use of aspirin (the most common antiplatelet prescribed) was 83.6%, 66.0%, and 73.3%, respectively, and the use of an ACE inhibitor was 58.1%, 42.8%, and 61.3%, respectively (Figures 1(a)–1(c)) [2]. In a Canadian study of more than 20,000 patients with stable CAD aged ≥65 years old, the use of optimal medical therapy (defined as a beta blocker, ARB/ACE inhibitor, and statin) was 61%. Aspirin could not be included in the analysis because of database limitations [34].

#### 3.2.2. Peripheral Artery Disease

In a Swedish national healthcare database analysis of 18,742 patients with intermittent claudication (IC) or critical limb ischemia (CLI), 59.8% of patients received statins and 57.0% an ARB/ACE inhibitor at baseline hospital admission for revascularization [35]. Two-thirds of patients received low-dose aspirin (7.9% as part of dual antiplatelet therapy) and 7.2% received clopidogrel (as per guidelines for lower-extremity PAD, either agent can be used; Supplementary [Supplementary-material supplementary-material-1]). Overall, only 65% of patients with IC and 45% of patients with CLI received best medical therapy (i.e., an antiplatelet or anticoagulant and a statin) at admission. Use of secondary preventive therapies did, however, improve after surgery, e.g., 80% of patients with CLI received aspirin within the first 3 months following revascularization [35]. For patients with PAD enrolled in the REACH registries of Canada, France, and Germany, use of statins, aspirin, and ACE inhibitors was lower than that observed for patients with CAD, whereas use of diabetic agents and oral anticoagulants was higher (Figures 1(a)–1(c)) [2]. A small single-center chart review of 42 patients with PAD and diabetes in Canada revealed that only 60% of patients were discharged on a combination of a statin, an ARB/ACE inhibitor, and an antiplatelet following vascular surgery. This proportion was higher in patients with concomitant CAD versus those with isolated PAD (78% and 46%, respectively) [36]. Indeed, several studies demonstrated that the use of therapy to prevent CV events is lower in patients with PAD than in patients with CAD (Figures 1(a)–1(c)).

### 3.3. Humanistic Burden

#### 3.3.1. Coronary Artery Disease

CAD underlies a spectrum of conditions dependent on the degrees of stenosis, the hemodynamic consequences of the stenosis, plaque characteristics, and the level of myocardial ischemia [23, 28]. Potential outcomes from CAD include major adverse CV events (MACE; CV death, MI, and stroke), heart failure, hospitalization, disability, and reduced activities of daily living (Table 1) [37–40].

**Figure 2:**
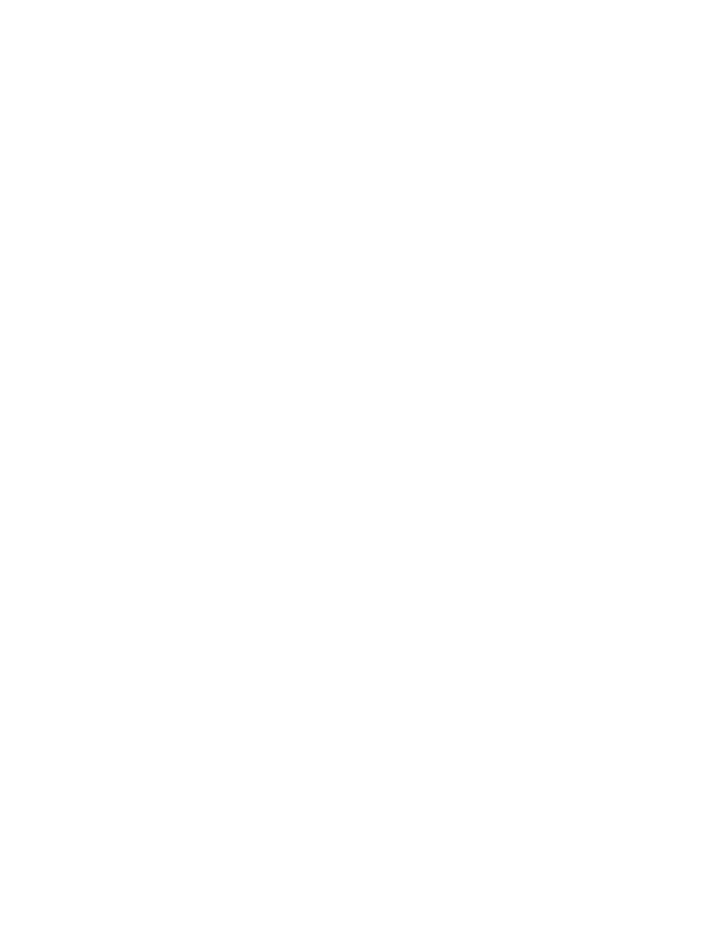
The persistent risk of MACE (a) up to 3 years following an index MI (adapted from Blin et al*.,* 2016) [3] and (b) up to 4.5 years following an index MI stratified by age (<72 years old versus ≥72 years old) and risk category (low versus high^a^) (adapted from Jernberg et al., 2015) [4]. ^a^High-risk patients were predefined as those with ≥1 of the following risk factors prior to index; MI: diabetes mellitus, at least one MI prior to index MI event, CABG (proxy for multi-vessel CAD), PAD, stroke, heart failure, or diagnosis of chronic renal dysfunction. CABG, coronary artery bypass graft; CAD, coronary artery disease; CV, cardiovascular; HR, high risk; LR, low risk; MACE, major adverse cardiovascular events; MI, myocardial infarction; PAD, peripheral artery disease.

MACE is a common outcome measure in studies of patients with CAD. In REACH Canada, 2.7% of patients with CAD had a MACE at 1-year follow-up; in datasets from France and Germany the incidence of MACE was ~6% at 2 years follow-up (Figures 1(a)–1(c)). It is well-established that the risk of MACE is elevated in patients with CAD and a previous CV event. Two studies demonstrated that the risk of MACE remains high for up to 4.5 years after an MI (Figures 2(a) and 2(b)) [3, 4].

Survival rates in patients with CAD have improved; for example, in the USA, age-adjusted mortality between 1980 and 2002 was approximately halved in both male (from 900 to 400 per 100,000) and female (from 500 to 250 per 100,000) patients with CAD [37]. Similarly, in Germany, age-adjusted mortality fell from ~300 to ~140 per 100,000 between 1990 and 2015 [41]. There has also been an increased focus on patients' overall quality of life. A large UK-based study highlighted that patients with CAD reported a lower quality of life and more depressive symptoms than participants in the healthy control group. The observed changes were gender-specific, with women reporting a higher quality of life than men in the 2 and 4 years following a CV event [39]. In Canada, 4305 (18.8%) of 22,917 patients with stable angina reported symptoms of depression. Patients with depression had a higher risk of death and admission for MI compared with those with CAD without depression [40]. Like CV comorbidities, musculoskeletal conditions are also frequent among patients with CAD, leading to substantial disability. In a cross-sectional observational study involving 11 hospitals in Canada, 1010 (56%) of 1803 patients with CAD had musculoskeletal conditions, with arthritis/joint pain accounting for 64.4% of these [42].

#### 3.3.2. Peripheral Artery Disease

Ischemia of the lower limbs can be classified as functional or critical. IC, the most frequent clinical presentation of PAD, represents functional ischemia in which blood flow may be sufficient at rest but insufficient during exercise [43]. Approximately 1 in 4 patients with IC will experience worsening of symptoms or the development of CLI or acute limb ischemia (ALI). These conditions are characterized by progressive (CLI) or sudden onset (ALI) of pain at rest or nonhealing ischemic wounds or gangrene [27, 44, 45] and can ultimately lead to a loss of limbs [46]. In the UK-based OXVASC study involving more than 90,000 general medical patients from primary care, CLI occurred at a rate of 22 events per 100,000 patients per year (mean age: 75.2 years) and ALI at a rate of 10 events per 100,000 patients per year (mean age: 76.3 years) [46]. In a retrospective analysis of US healthcare insurer databases, the mean incidence of CLI was 0.35% per year (in a population with a mean age of ~69 years), representing 11.08% of patients with PAD [18].

PAD has been widely associated with a reduced health-related quality of life (HRQoL), predominantly because of the functional limitations caused by symptoms and the consequences of PAD (e.g., pain, functional loss, and loss of limbs) [43]. Patients who self-reported a physician diagnosis of PAD in the EU and the US National Health and Wellness Survey had significantly lower mental and physical HRQoL scores compared with the general population in adjusted analyses [43]. In a single-center US study, patients with asymptomatic PAD or atypical symptoms had greater mobility decline than those without PAD or with symptoms of IC, with a significantly decreased ability to walk for 6 minutes continuously [47]. In a Swedish longitudinal cohort study at two vascular clinics, 64 (29%) of 219 patients scheduled for treatment of IC or CLI reported chronic widespread pain, which was strongly associated with a reduced HRQoL at baseline and 12 months after treatment [48].

PAD is also associated with an increased risk of MACE. Findings from the REACH registries revealed similar to higher event rates for CV death in patients with any PAD than in those with any CAD. This was also true for the combined outcomes of MACE and MACE plus hospitalization (Figures 2(a) and 2(b)) [1, 2]. Blin et al. demonstrated that PAD was the most significant factor in predicting the risk of reinfarction/stroke/transient ischemic attack within a year of MI (present in 22.9% of patients who had an event versus 11.4% of patients who did not;* P *= 0.0005) [3]. Data on mortality rates in patients with isolated PAD were sparse, but in the PREPARED-UK registry, the 2-year mortality rate among patients with IC was 8.4% [49]. One-year mortality rates in patients with IC and CLI identified in the Swedish national healthcare registry were 1.9% and 11.7%, respectively (approximately one-third of patients had concomitant CAD) [35].

### 3.4. Economic Burden

#### 3.4.1. Coronary Artery Disease

Without appropriate treatment and prevention, the silent and progressive nature of atherosclerosis ultimately leads to CV events, hospitalization, and revascularization procedures, which represent a significant economic burden. These include hospitalization and medication costs and loss of productivity [1–3].

MI or stroke can require lengthy hospital stays: one French cohort study reported a mean length of stay of 7 days for patients hospitalized for MI [3]. The average direct medical costs associated with hospitalization for CAD were reported from the REACH registries in France, Germany [2], and Canada [1]. In France, the average direct 1- and 2-year costs per CAD patient amounted to €1122 and €1746, respectively. In Germany, the equivalent costs were €1042 and €1784, respectively. Most of the direct costs could be attributed to cardiac surgery or intervention, with the most expensive procedure costing €17,626 (for CABG combined with coronary angioplasty/stenting) [2]. In Canada, mean hospitalization costs for patients with CAD were C$1743 per patient (C$2.2 billion overall, for all patients with CAD at the national level) and the average annual medication costs were C$1593 per patient. Total average annual hospitalization costs significantly increased as the number of affected arterial beds increased (C$380, C$1403, and C$3465 for 0, 1, and 2–3 affected arterial beds, respectively) [1]. A US study showed that, during the first 12 months after an acute coronary syndrome (ACS), patients incurred an additional $31,061 (*P* < 0.001) in total healthcare expenditures compared with those without ACS [50]. One study estimated lifetime costs of revascularization by CABG or PCI in the USA at $196,256 and $187,532 per patient, respectively [51].

#### 3.4.2. Peripheral Artery Disease

The direct costs associated with PAD are even higher than those for CAD because of the higher rates of polyvascular disease and higher number of annual CV events and hospitalization rates [52]. According to data collected in the REACH registry, the average cumulative 1- and 2-year costs associated with hospitalizations for vascular reasons in France amounted to €1994 and €3182 per PAD patient, respectively. In Germany, the equivalent 2-year cost was €2724. Peripheral revascularizations accounted for approximately half of these costs [2]. Similarly, in Canada, mean hospitalization costs were more than three times higher for patients with PAD than those with CAD (C$4677 and C$1743 per patient, respectively). Annual medication costs were only slightly higher for patients with PAD than those with CAD (C$1695 and C$1593 per patient, respectively) [1]. In the USA, the total PAD-related annualized healthcare cost in the symptomatic PAD patient population was $4006 per patient [53].

#### 3.4.3. Indirect Costs of Coronary Artery Disease and Peripheral Artery Disease

In addition to these direct costs, CAD and PAD may lead to substantial morbidity- and mortality-related productivity costs [54]. In Canada, the per-patient cost of absenteeism after MI ranged from C$6974 at 1-year post MI to C$10,702 at 4 years. Annual presenteeism costs (reduced productivity at work because of illness) for the whole US population were estimated at US$43.3 billion for heart disease and US$9.5 billion for stroke, whereas annual absenteeism costs were estimated at US$11.9 billion for coronary heart disease and US$2.2 billion for stroke [54]. The 2010 National Health and Wellness Surveys in the EU and the USA reported significant impairment in work in patients with PAD, based on absenteeism, presenteeism, overall work productivity loss, and activity impairment [43].

## 4. Conclusions

CAD and PAD represent a substantial medical and economic burden worldwide. Although some progress has been made to improve survival, morbidity, quality of life, and the direct costs of atherothrombotic disease are high and are increasing. Outcomes could be improved with greater use of guideline-recommended antithrombotics and drugs/lifestyle changes to control modifiable CV risk factors.

## Figures and Tables

**Table 1:** Clinical burden of CAD.

	Smolderen 2012^a^	Smolderen 2012^a^	Smolderen 2010^b^
Country	France	Germany	Canada
Condition	CAD	CAD	CAD
Registry	REACH	REACH	REACH
Cohort size	2473	3510	1362
Age (mean)	69.1	68.0	68.3
Non-fatal events (per 1000 patients)			
Non-fatal stroke	24.0	29.5	8.1
TIA	8.4	17.2	6.6
Non-fatal MI	20.4	17.5	9.6
Unstable angina	49.2	102.3	39.8
CHF	46.1	64.2	14.0
CABG	8.4	27.8	19.2
PCI/Stenting	55.0	84.7	29.5

^a^Event rates per 1000 patients over 2 years.

^b^Event rates per 100 patients over 1 year.

CABG, coronary artery bypass grafting; CAD, coronary artery disease; CHF, chronic heart failure; MI, myocardial infarction; PCI, percutaneous coronary intervention; TIA, transient ischemic attack; REACH, Reduction of Atherothrombosis for Continued Health.

## Supplementary Materials

Supplementary Materials The supplementary materials contain the inclusion and exclusion criteria, a summary of the guideline recommendations on the use of antiplatelet therapy in patients with CAD or PAD, and the PRISMA diagram of the literature search for this systematic review.

## References

[1] Smolderen K. G., Bell A., Lei Y. (2010). One-year costs associated with cardiovascular disease in Canada: insights from the REduction of Atherothrombosis for Continued Health (REACH) registry. *Canadian Journal of Cardiology*.

[2] Smolderen K. G., Wang K., De Pouvourville G. (2012). Two-year vascular hospitalisation rates and associated costs in patients at risk of atherothrombosis in France and Germany: Highest burden for peripheral arterial disease. *European Journal of Vascular and Endovascular Surgery*.

[3] Blin P., Philippe F., Bouee S. (2016). Outcomes following acute hospitalised myocardial infarction in France: an insurance claims database analysis. *International Journal of Cardiology*.

[4] Jernberg T., Hasvold P., Henriksson M., Hjelm H., Thuresson M., Janzon M. (2015). Cardiovascular risk in post-myocardial infarction patients: Nationwide real world data demonstrate the importance of a long-term perspective. *European Heart Journal*.

[5] Libby P. (2013). Mechanisms of acute coronary syndromes and their implications for therapy. *The New England Journal of Medicine*.

[6] Hiatt W. R., Armstrong E. J., Larson C. J., Brass E. P. (2015). Pathogenesis of the limb manifestations and exercise limitations in peripheral artery disease. *Circulation Research*.

[7] Libby P., Ridker P. M., Hansson G. K. (2011). Progress and challenges in translating the biology of atherosclerosis. *Nature*.

[8] Bauersachs R., Zannad F. (2018). Rivaroxaban: a new treatment paradigm in the setting of vascular protection?. *Thrombosis and Haemostasis*.

[9] Bhatt D. L., Steg P. G., Ohman E. M. (2006). International prevalence, recognition, and treatment of cardiovascular risk factors in outpatients with atherothrombosis. *Journal of the American Medical Association*.

[10] Steg P. G., Bhatt D. L., Wilson P. W. (2007). One-year cardiovascular event rates in outpatients with atherothrombosis. *Journal of the American Medical Association*.

[11] Cortés-Beringola A., Fitzsimons D., Pelliccia A., Moreno G., Martín-Asenjo R., Bueno H. (2017). Planning secondary prevention: room for improvement. *European Journal of Preventive Cardiology*.

[12] GBD 2016 DALYs and HALE Collaborators (2017). Global, regional, and national disability-adjusted life-years (DALYs) for 333 diseases and injuries and healthy life expectancy (HALE) for 195 countries and territories, 1990-2016: a systematic analysis for the global burden of disease study 2016. *Lancet*.

[13] Fowkes F. G. R., Aboyans V., Fowkes F. J. I., McDermott M. M., Sampson U. K. A., Criqui M. H. (2017). Peripheral artery disease: Epidemiology and global perspectives. *Nature Reviews Cardiology*.

[14] GBD Disease Injury Incidence Prevalence Collaborators (2017). Global, regional, and national incidence, prevalence, and years lived with disability for 328 diseases and injuries for 195 countries, 1990-2016: a systematic analysis for the global burden of disease study 2016. *Lancet*.

[15] Gowdak L. H. W. (2017). Prevalence of refractory angina in clinical practice. *Heart and Metabolism*.

[16] Puymirat É. (2015). Épidémiologie de la maladie coronaire [Epidemiology of coronary artery disease]. *La Revue Du Praticien*.

[17] Selvin E., Erlinger T. P. (2004). Prevalence of and risk factors for peripheral arterial disease in the United States: results from the National Health and Nutrition Examination Survey, 1999-2000. *Circulation*.

[18] Nehler M. R., Duval S., Diao L. (2014). Epidemiology of peripheral arterial disease and critical limb ischemia in an insured national population. *Journal of Vascular Surgery*.

[19] Hong Y., Sebastianski M., Makowsky M., Tsuyuki R., McMurtry M. S. (2016). Administrative data are not sensitive for the detection of peripheral artery disease in the community. *Vascular Medicine (United Kingdom)*.

[20] Diehm C., Schuster A., Allenberg J. R. (2004). High prevalence of peripheral arterial disease and co-morbidity in 6880 primary care patients: cross-sectional study. *Atherosclerosis*.

[21] Olinic D. M., Spinu M., Olinic M. (2018). Epidemiology of peripheral artery disease in Europe: VAS educational paper. *International Angiology*.

[22] Sadrzadeh Rafie A. H., Stefanick M. L., Sims S. T. (2010). Sex differences in the prevalence of peripheral artery disease in patients undergoing coronary catheterization. *Vascular Medicine*.

[23] Montalescot G., Sechtem U., Achenbach S. (2013). ESC guidelines on the management of stable coronary artery disease: the task force on the management of stable coronary artery disease of the european society of cardiology. *European Heart Journal*.

[24] Aboyans V., Ricco J. B., Bartelink M. E. L. (2018). ESC guidelines on the diagnosis and treatment of peripheral arterial diseases, in collaboration with the European Society for Vascular Surgery (ESVS): document covering atherosclerotic disease of extracranial carotid and vertebral, mesenteric, renal, upper and lower extremity arteries. *European Heart Journal*.

[25] Mancini G. B. J., Gosselin G., Chow B. (2014). Canadian Cardiovascular Society Guidelines for the diagnosis and management of stable ischemic heart disease. *Canadian Journal of Cardiology*.

[26] Abramson B. L., Huckell V. (2005). *Canadian Cardiovascular Society 2005 peripheral artery disease consensus document*.

[27] Gerhard-Herman M. D., Gornik H. L., Barrett C. (2016). AHA/ACC guideline on the management of patients with lower extremity peripheral artery disease: a report of the american college of cardiology/american heart association task force on clinical practice guidelines. *JACC: Journal of the American College of Cardiology*.

[28] Fihn S. D., Gardin J. M., Abrams J. (2012). Guideline for the diagnosis and management of patients with stable ischemic heart disease: a report of the american college of cardiology foundation/american heart association task force on practice guidelines, and the american college of physicians, american association for thoracic surgery, preventive cardiovascular nurses association, society for cardiovascular angiography and interventions, and society of thoracic surgeons. *Circulation*.

[29] Haute Autorité de Santé Maladie coronarienne stable [Stable coronary artery disease]. https://www.has-sante.fr/portail/upload/docs/application/pdf/liste_ald_maladie_coronarienne.pdf.

[30] Haute Autorité de Santé Artériopathie oblitérante des membres inférieurs [Obliterative arteriopathy of the lower limbs]. https://www.has-sante.fr/portail/upload/docs/application/pdf/ald3_aomi_guide_cardiovasc_post_corrlemire_revuenp28avril__205.pdf.

[31] National Institutes of Health Stable angina: management. https://www.nice.org.uk/guidance/cg126/resources/stable-angina-management-pdf-35109453262021.

[32] Guidelines Network Management of stable angina: a national clinical guideline 96. https://www.sign.ac.uk/assets/sign96.pdf.

[33] Ferrari R., Abergel H., Ford I. (2013). Gender- and age-related differences in clinical presentation and management of outpatients with stable coronary artery disease. *International Journal of Cardiology*.

[34] Chun S., Qiu F., Austin P. C. (2015). Predictors and outcomes of routine versus optimal medical therapy in stable coronary heart disease. *American Journal of Cardiology*.

[35] Sigvant B., Kragsterman B., Falkenberg M. (2016). Contemporary cardiovascular risk and secondary preventive drug treatment patterns in peripheral artery disease patients undergoing revascularization. *Journal of Vascular Surgery*.

[36] Sunderland M., De Jong M., Bates D. (2013). Vascular protection in patients with diabetes admitted for vascular surgery in a canadian tertiary care hospital: pilot study. *The Canadian Journal of Hospital Pharmacy*.

[37] Steg P. G., Dorman S. H. (2011). The evolving nature of coronary artery disease. *European Heart Journal Supplements*.

[38] Ayoub C., Bernick J., Arasaratnam P. (2016). Coronary artery disease in french canadians—investigation of a suggested vulnerable population. *Canadian Journal of Cardiology*.

[39] Zaninotto P., Sacker A., Breeze E., Mcmunn A., Steptoe A. (2016). Gender-specific changes in well-being in older people with coronary heart disease: evidence from the english longitudinal study of ageing. *Aging & Mental Health*.

[40] Szpakowski N., Bennell M. C., Qiu F. (2016). Clinical impact of subsequent depression in patients with a new diagnosis of stable angina: a population-based study. *Circulation: Cardiovascular Quality and Outcomes*.

[41] Deutsche Herzstiftung e.V. Deutscher Herzbericht 2017. https://www.herzstiftung.de/herzbericht.

[42] Marzolini S., Oh P. I., Alter D. (2012). Musculoskeletal comorbidities in cardiac patients: prevalence, predictors, and health services utilization. *Archives of Physical Medicine and Rehabilitation*.

[43] Marrett E., DiBonaventura M. D., Zhang Q. (2013). Burden of peripheral arterial disease in europe and the united states: a patient survey. *Health and Quality of Life Outcomes*.

[44] Norgren L., Hiatt W. R., Dormandy J. A. (2007). Inter-society consensus for the management of peripheral arterial disease (TASC II). *Journal of Vascular Surgery*.

[45] Hirsch A. T., Haskal Z. J., Hertzer N. R. (2006). ACC/AHA 2005 practice guidelines for the management of patients with peripheral arterial disease (lower extremity, renal, mesenteric, and abdominal aortic): a collaborative report from the american association for vascular surgery/society for vascular surgery, society for cardiovascular angiography and interventions, society for vascular medicine and biology, society of interventional radiology, and the acc/aha task force on practice guidelines (writing committee to develop guidelines for the management of patients with peripheral arterial disease): endorsed by the american association of cardiovascular and pulmonary rehabilitation; national heart, lung, and blood institute; society for vascular nursing; transatlantic inter-society consensus; and vascular disease foundation. *Journal of the American College of Cardiology*.

[46] Howard D. P. J., Banerjee A., Fairhead J. F., Hands L., Silver L. E., Rothwell P. M. (2015). Population-based study of incidence, risk factors, outcome, and prognosis of ischemic peripheral arterial events: implications for prevention. *Circulation*.

[47] McDermott M. M., Ferrucci L., Liu K. (2010). Leg symptom categories and rates of mobility decline in peripheral arterial disease. *Journal of the American Geriatrics Society*.

[48] Lindgren H., Gottsater A., Qvarfordt P., Bergman S. (2016). All cause chronic widespread pain is common in patients with symptomatic peripheral arterial disease and is associated with reduced health related quality of life. *European Journal of Vascular and Endovascular Surgery*.

[49] Stansby G., Mister R., Fowkes G. (2011). High risk of peripheral arterial disease in the United Kingdom: 2-year results of a prospective registry. *Angiology*.

[50] Johnston S. S., Curkendall S., Makenbaeva D. (2011). The direct and indirect cost burden of acute coronary syndrome. *Journal of Occupational and Environmental Medicine*.

[51] Zhang Z., Kolm P., Grau-Sepulveda M. V. (2015). Cost-effectiveness of revascularization strategies: the ASCERT study. *JACC: Journal of the American College of Cardiology*.

[52] Mahoney E. M., Wang K., Keo H. H. (2010). Vascular hospitalization rates and costs in patients with peripheral artery disease in the United States. *Circulation: Cardiovascular Quality and Outcomes*.

[53] Chase M. R., Friedman H. S., Navaratnam P., Heithoff K., Simpson Jr. R. J. (2016). Comparative assessment of medical resource use and costs associated with patients with symptomatic peripheral artery disease in the United States. *Journal of Managed Care and Specialty Pharmacy*.

[54] Gordois A. L., Toth P. P., Quek R. G. (2016). Productivity losses associated with cardiovascular disease: a systematic review. *Expert Review of Pharmacoeconomics & Outcomes*.

