# Supplementary material for: Burden of Coronary Artery Disease and Peripheral Artery Disease: A Literature Review
Source: Cardiovasc Ther. 2019 Nov 26;2019:8295054. doi: 10.1155/2019/8295054 (PMC7024142; doi:10.1155/2019/8295054)
Supplement: Supplementary Materials — The supplementary materials contain the inclusion and exclusion criteria, a summary of the guideline recommendations on the use of antiplatelet therapy in patients with CAD or PAD, and the PRISMA diagram of the literature search for this systematic review. [file 8295054.f1.docx]

Supplementary material

**SUPPLEMENTARY TABLE 1** Inclusion and exclusion criteria

|  | **Inclusion criteria** | **Exclusion criteria** |
| --- | --- | --- |
| Population (P) | - Adult patients with CAD  and PAD^a^ | - Patients, children, adolescents without CAD or PAD |
| Interventions (I) | - No intervention specified | - Not applicable |
| Comparators (C) | - No comparators specified | - Not applicable |
| Outcomes (O) | - Epidemiology (incidence and prevalence) - Disease and complication risk factors - Treatment pattern (clinical guidelines) - Humanistic burden (QoL patient satisfaction, mortality, and morbidity) - Economic burden (resource use including hospitalizations, cost, and absenteeism) | - Other outcomes |
| Study design (S) | - Observational studies - Reviews | - Other studies |

^a^The geographic scope of the review included Canada, France, Germany, Sweden, the UK and the USA

CAD, coronary artery disease; PAD, peripheral artery disease; QoL, quality of life

**SUPPLEMENTARY TABLE 2** Guideline recommendations on the use of antiplatelet therapy in patients with stable angina/stable CAD

| Guideline/Country | Population | Recommendations |
| --- | --- | --- |
| ESC (2013)^1^  EU | Patients with stable CAD | - Long-term low-dose aspirin (75–150 mg per day), or clopidogrel in case of aspirin intolerance, in all patients with established CAD - DAPT is not routinely recommended - DAPT is indicated after BMS implantation for at least 1 month - DAPT is indicated for 6–12 months after implantation of second-generation DES |
| HAS, (2016a)^2^  France | Patients with stable CAD | - Long-term low-dose aspirin (75–160 mg per day) as monotherapy, or:   - Aspirin (75–160 mg per day) + clopidogrel (75 mg per day) post MI for 1 year   - Aspirin (75–160 mg per day) + prasugrel (100 mg per day) post MI for 1 year   - Aspirin (75–160 mg per day) + ticagrelor (180 mg per day) post MI for 1 year |
| NICE, (2011 updated in 2016)^3^  UK | Patients with stable angina | - Long-term low-dose aspirin (75 per day) |
| SIGN (2007)^a,4^  Scotland | Patients with stable angina | - Long-term aspirin (75–150 mg per day) |
| ACCF/AHA/ACP/AATS/PCNA/SCAI/STS (2012)^5^  USA | Patients with stable ischemic heart disease | - Aspirin (75–162 mg per day) continued indefinitely in the absence of contraindications, or clopidogrel when aspirin is contraindicated - Treatment with aspirin (75–162 mg per day) and clopidogrel (75 mg per daily) might be reasonable in certain high-risk patients |
| ACC/AHA (2016)^6^  USA | Patients with SIHD | - DAPT for a minimum of 1 month after BMS implantation - DAPT for a minimum of 6 months in patients with stable CAD treated with DES implantation (3 months may be reasonable in patients with a high risk of bleeding or who develop significant overt bleeding) - DAPT for more than 12 months for an MI within the previous 1–3 years may be reasonable, but only in patients at low bleeding risk - DAPT for 12 months after CABG may be reasonable to improve vein graft patency - In patients treated with DAPT, aspirin 81 mg per day (ranging from 75 mg to 100 mg per day) is recommended alongside clopidogrel - DAPT is not beneficial in patients without a history of ACS, coronary stent implantation, or CABG ≤12 months |
| CCS (2014)^7^  Canada | Patients with SIHD | - Aspirin (81 mg per day) or clopidogrel (75 mg per day) in case of aspirin intolerance - DAPT should not be used in routine management of SIHD or beyond the time period required as a result of stenting |

^a^An update to these guidelines was published in April 2018 (SIGN151)
ACC, American College of Cardiology; AATS, American Association for Thoracic Surgery; ACCF, American College of Cardiology Foundation; ACP, American College of Physicians; AHA, American Heart Association; BMS, bare-metal stent; CABG, coronary artery bypass grafting; CAD, coronary artery disease; CCS, Canadian Cardiology Society; DAPT, dual antiplatelet therapy; DES, drug-eluting stent; ESC, European Society of Cardiology; HAS, National Authority for Health; MI, myocardial infarction; NICE, National Institute for Health and Care Excellence; PCNA, Preventive Cardiovascular Nurses Association; SCAI, Society for Cardiovascular Angiography and Interventions; SIGN, Scottish Intercollegiate Guidelines Network; SIHD, stable ischemic heart disease; STS, Society of Thoracic Surgeons

**Supplementary Table 3** Guideline recommendations on the use of antiplatelet therapy in patients with PAD

| Guideline/Country | Population | Recommendations |
| --- | --- | --- |
| ESC (2017)  EU | Symptomatic carotid stenosis | - Long-term single antiplatelet therapy, aspirin (75–100 mg per day) or clopidogrel (75 mg per day) as an alternative in patients with aspirin intolerance - DAPT with aspirin and clopidogrel is recommended for at least 1 month after CAS |
|  | Symptomatic patients with LEAD | - Long-term single antiplatelet therapy, aspirin (75–100 mg per day) or clopidogrel (75 mg per day) in symptomatic patients, in all patients who have undergone revascularization, and after infra-inguinal bypass surgery - In patients requiring antiplatelet therapy, clopidogrel may be preferred over aspirin |
|  | Patients with PAD and CAD | - DAPT may be prolonged beyond 1 month when there is a prior history (<1 year) of ACS and/or percutaneous coronary intervention |
| HAS (2016b)  France | Patients with LEAD | - Lifelong aspirin therapy (75–325 mg per day) or clopidogrel (75 mg per day) in case of aspirin intolerance |
| AHA/ACC (2016)  USA^8^ | Patients with LEAD | - Aspirin alone (75–325 mg per day) or clopidogrel alone (75 mg per day) - DAPT (aspirin and clopidogrel) may be reasonable to reduce the risk of limb-related events in patients with symptomatic PAD after lower extremity revascularization - Cilostazol to improve symptoms and increase walking distance in patients with claudication |
| CCS (2005)  Canada | Patients with symptomatic PAD | - Lifelong aspirin therapy (75–325 mg per day) or clopidogrel (75 mg per day) in case of aspirin intolerance |
| CCS (2011)  Canada | Patients with symptomatic PAD | - Low-dose aspirin (75–162 mg per day) or clopidogrel (75 mg per day) providing the bleeding risk is low (Class IIb, Level B) - Choice of drug may depend on patient preference and cost considerations |
|  | Patients with symptomatic PAD with overt CAD or CeVD | - Antiplatelet therapy as indicated for CAD and/or CeVD (Class I, Level A) |

ACC, American College of Cardiology; ACS, acute coronary syndrome; AHA, American Heart Association; CAD, coronary artery disease; CAS, carotid artery stenosis; CCS, Canadian Cardiology Society; CeVD, cerebrovascular disease; DAPT, dual antiplatelet therapy; ESC, European Society of Cardiology; HAS, National Authority for Health; LEAD, lower extremity artery disease; PAD, peripheral artery disease


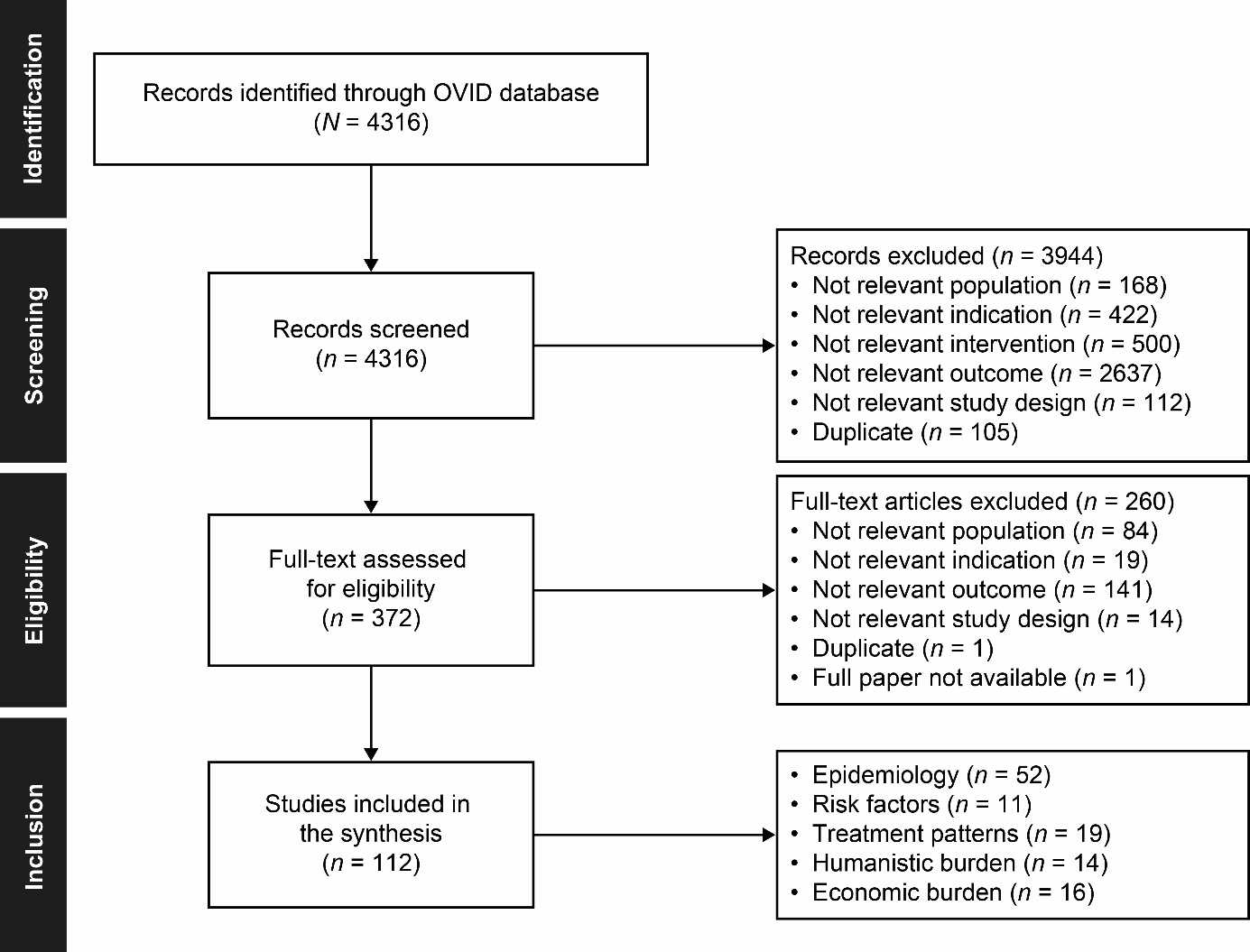


**SUPPLEMENTARY FIGURE 1** PRISMA diagram of the search

References

1. Montalescot G, Sechtem U, Achenbach S, et al. 2013 ESC guidelines on the management of stable coronary artery disease: the task force on the management of stable coronary artery disease of the European Society of Cardiology. *Eur Heart J*. 2013;34:2949–3003.

2. Haute Autorité de Santé. Artériopathie oblitérante des membres inférieurs [Obliterative arteriopathy of the lower limbs]. <https://www.has-sante.fr/portail/upload/docs/application/pdf/ald3_aomi_guide_cardiovasc_post_corrlemire_revuenp28avril__205.pdf>. Accessed 22 January 2019.

3. National Institutes of Health. Stable angina: management. <https://www.nice.org.uk/guidance/cg126/resources/stable-angina-management-pdf-35109453262021>. Accessed 22 January 2019.

4. Scottish Intercollegiate Guidelines Network. Management of stable angina: a national clinical guideline 96. <https://www.sign.ac.uk/assets/sign96.pdf>. Accessed 22 January 2019.

5. Fihn SD, Gardin JM, Abrams J, et al. 2012 ACCF/AHA/ACP/AATS/PCNA/SCAI/STS guideline for the diagnosis and management of patients with stable ischemic heart disease: a report of the American College of Cardiology Foundation/American Heart Association task force on practice guidelines, and the American College of Physicians, American Association for Thoracic Surgery, Preventive Cardiovascular Nurses Association, Society for Cardiovascular Angiography and Interventions, and Society of Thoracic Surgeons. *Circulation*. 2012;126:e354–e471.

6. Levine GN, Bates ER, Bittl JA, et al. 2016 ACC/AHA guideline focused update on duration of dual antiplatelet therapy in patients with coronary artery disease: a report of the American College of Cardiology/American Heart Association Task Force on Clinical Practice Guidelines. *J Am Coll Cardiol*. 2016;68:1082–1115.

7. Mancini GB, Gosselin G, Chow B, et al. Canadian Cardiovascular Society guidelines for the diagnosis and management of stable ischemic heart disease. *Can J Cardiol*. 2014;30:837–849.

8. Gerhard-Herman MD, Gornik HL, Barrett C, et al. 2016 AHA/ACC guideline on the management of patients with lower extremity peripheral artery disease: A report of the American College of Cardiology/American Heart Association Task Force on Clinical Practice Guidelines. *J Am Coll Cardiol*. 2017;69:e71–e126.
